# St. George's Hospital

**Published:** 1907-05-11

**Authors:** 


### St. George's Hospital.

A special court of the Governors was held on the 3rd instant, to once more consider the question of the expediency of substituting an elected house committee for the old open board. Mr. West, as treasurer of the hospital, moved a resolution to set up an elected house committee, to delete certain of the laws, and to pass others constituting an elected committee. In order, as far as possible, to meet the reasonable prejudices of the older men, Mr. West proposed that Governors who are not members of the house committee shall be entitled to be present at its meetings, to join in any debate, but not to vote. Mr. West was supported by Mr. H. Claude Hay, the Hon. D. Fortescue and Mr. R. H. C. Harrison, who have rendered good service in the cause of reform. After a strenuous opposition, led by Lord Frederick Fitzroy, Captain Farmer and Mr. C. G. Heathcote, Mr. West's resolution creating a house committee was eventually carried by thirty votes to five. The thanks of the Governors especially, and also of the public who take an interest in hospitals, are due to Mr. A. William West, whose services to St. George's Hospital, in this and many other ways, entitle him to generous recognition. We congratulate Mr. West upon the success which has crowned his continuous efforts in the cause of reform, and we are glad to realise that he is young enough to render it probable that, he will live to see the results of his efforts, in the erection of a new and modern hospital, either on the preent site, or in a portion of the city where it is likely to render even greater service, within the next ten years.

Now that the Governors of St. George's Hospital have put themselves in line with modern developments, and have taken a step which is sure to commend itself to the giving public, St. George's Hospital should go forward and prosper greatly. The King's and other Funds will no doubt liberally support the policy of Mr. West, and the introduction of an energetic, up to date, businesslike administration, into the affairs of St. George's Hospital, must bring with it renewed energy and confidence. Money should now flow into its exchequer; new supporters should come forward in large numbers, not only with their money, but with the offer of personal service, so that the new house committee may consist of younger and more active men, who are prepared to give time and brains to the affairs of the institution.

We hope that St. George's Hospital may now, in due course, sell its present site, and emigrate to a district where the population urgently need the hospital care which it can afford. As we understand Mr. West's proposals, they will follow the lines of the report of the sub-committee which sat for over six months, and, by eliciting all the facts, make it possible to evolve a plan upon which St. George's Hospital may readily be developed upon modern lines. That plan will entail the sale of the site which ought to realise something like £400,000, in which case the sum which the public may be asked to give is not likely to be unreasonably large. Having placed the management of its affairs upon a business basis by the institution of an elected house committee, new life should be brought into the administration, which cannot fail to produce momentous results. We are confident that the profession and the public are in agreement that, the proper course for St. George's Hospital to adopt is, to secure a new hospital upon an adequate site, with all reasonable despatch. This being so, the sooner this policy is publicly announced, the sooner will the permanent income of the hospital be increased, and the whole of its business affairs placed upon a sound basis.

